# Autologous and synthetic vascular grafts trigger different host responses in the anastomotic regions and in the perivascular adipose tissue during the early healing phase

**DOI:** 10.1016/j.mtbio.2025.102089

**Published:** 2025-07-15

**Authors:** Sabrina Rohringer, Sophie Johanna Specht, Anna-Maria Schmitt, Selin Topcu, Karl Heinrich Schneider, Marjan Enayati, Christian Grasl, Katharina Ehrmann, Stefan Baudis, Herbert Kiss, Heinrich Schima, Bruno Karl Podesser, Helga Bergmeister

**Affiliations:** a Center for Biomedical Research and Translational Surgery, Medical University of Vienna, Waehringer Guertel 18-20, 1090, Vienna, Austria; b Ludwig Boltzmann Institute for Cardiovascular Research, Waehringer Guertel 18-20, 1090, Vienna, Austria; c Austrian Cluster for Tissue Regeneration, Donaueschingenstraße 13, 1200, Vienna, Austria; d Center for Medical Physics and Biomedical Engineering, Medical University of Vienna, Waehringer Guertel 18-20, 1090, Vienna, Austria; e Institute of Applied Synthetic Chemistry, TU Wien, Getreidemarkt 9/163, 1060, Vienna, Austria; f Department of Obstetrics and Gynecology, Division of Obstetrics and Feto-Maternal Medicine, Medical University of Vienna, Waehringer Guertel 18-20, 1090, Vienna, Austria

**Keywords:** Small-diameter vascular grafts, Graft healing, Anastomosis, Perivascular adipose tissue

## Abstract

Interactions between tissue and material post-implantation significantly impact the long-term behavior of vascular prostheses. Autologous materials are preferred for replacing small-diameter blood vessels due to their superior healing properties compared to synthetic options. This study aimed to characterize the host response to autologous and various synthetic grafts *in vitro* and *in vivo* during the early healing phase, focusing on anastomotic regions and perivascular adipose tissue (PVAT).

Immune cell activation by seeding human macrophages and neutrophils onto decellularized umbilical cord arteries (dUCAs), biodegradable thermoplastic polyether-urethane (TPU/TPUU), and expanded polytetrafluoroethylene (ePTFE) grafts was assessed *in vitro*. Autologous aortic grafts, TPU/TPUU, and ePTFE grafts were implanted in rats to evaluate cytokine expression in anastomotic regions at 24 h and 1 week, alongside protein and gene expression analyses of PVAT.

Decellularized UCA grafts elicited less pro-inflammatory macrophage activation than ePTFE and TPU/TPUU *in vitro*. Autologous grafts exhibited no inflammatory response in anastomotic regions, while ePTFE displayed significantly higher cytokine and pro-inflammatory gene expression at 24 h after implantation. TPU/TPUU specimens showed a delayed response. PVAT analyses revealed significant inflammation in ePTFE implants, contrasting with limited pro-inflammatory activation in autologous and TPU/TPUU grafts.

Significant differences in the upregulation of inflammatory cytokines and pro-inflammatory genes can be observed in the early postoperative healing phase. Therefore, new synthetic materials or therapeutic approaches targeting inadequate healing response at the early stage would be mandatory to overcome current graft performance limitations. In particular, the role of PVAT in vascular graft healing should be evaluated in detail.

## Highlights

• Significant differences in the early post-operative healing phase and inflammation were observed.
• Synthetic vascular grafts induced a significant inflammatory reaction in anastomotic regions.
• EPTFE showed a significant pro-inflammatory activation in the perivascular adipose tissue.

## Graphical abstract

**Figure:**
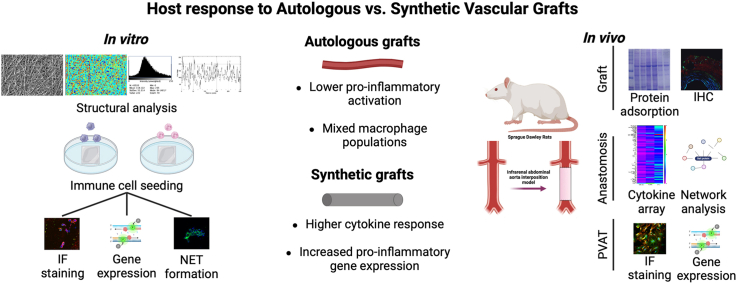

## 1 Introduction

In the early 1960s, surgeons developed a variety of coronary artery bypass graft (CABG) procedures using different blood vessels such as the internal thoracic and radial artery. René Gerónimo Favaloro was the first to use a free autologous saphenous vein graft (SVG) for an end-to-end anastomosis between the distal right coronary artery and the ascending aorta in 1967 [1]. His technique was adopted worldwide for coronary artery bypass grafting, and autologous veins and arteries remained the gold standard for small-diameter blood vessel replacement [[2], [3], [4]]. Impaired healing of autologous vessels is caused by abnormal hemodynamic stress, size and compliance mismatch at the anastomosis, patient condition [5] and vascular graft infection [6] during surgical intervention. Gaudino et al. (2017) showed that risk factors for impaired graft patency also include technical factors during implantation, such as the anastomosis technique, the vasodilatation protocol, the graft storage solution and the harvesting method [7].

Extensive research has been conducted to develop synthetic alternatives for patients with limited vascular availability due to comorbidities or previous vessel harvest. However, the clinical transition of synthetic vascular graft materials has so far not been achieved [8]. Synthetic vascular grafts made of expanded polytetrafluoroethylene (ePTFE) or polyethylene terephthalate (PET) are used for large blood vessel reconstruction but have a patency rate of less than 50 % over five years for small-diameter applications [9,10]. Prosthesis failure is mainly caused by a persistent foreign body reaction and the lack of a confluent endothelium, especially in the mid-graft area [8]. Both can be traced back to the limited biocompatibility of the graft material, which causes inflammation in the early phase and leads to persistent foreign body reactions and fibrous encapsulation after months and years of implantation [11]. Multiple studies investigated surface modifications of ePTFE to improve endothelialization and graft patency [[12], [13], [14], [15], [16], [17], [18]]. One of the main alternatives to ePTFE and PET is thermoplastic polyurethane (TPU) due to its excellent and customizable biomechanical properties and biocompatibility. However, the clinical application of TPU grafts is limited due to the undesired degradation of the material, which often results in aneurysmal dilatation [19] and potential toxic aromatic byproducts [20,21].

An important factor for successful graft healing and high patency rates, regardless of the material used, is the resolution of acute inflammation several days after implantation to avoid chronic inflammatory reactions [22,23]. The injury caused by the implantation of a biomaterial is the first step in the inflammatory cascade. Proteins are adsorbed on the material surface and contact between the blood and the material leads to further accumulation and migration of neutrophils. A provisional matrix forms, enabling the infiltration of macrophages that mediate phagocytosis. After a few days of implantation, adaptive immune cells migrate to the site of injury and begin to express factors that induce the polarization of macrophages into an anti-inflammatory phenotype [24,25].

In addition to an initial inflammatory response and its resolution, perivascular adipose tissue (PVAT) has been reported to be an important factor for vascular graft patency [26,27]. SVGs harvested with the non-touch technique preserving PVAT showed higher patency rates (83 %) over an implantation period of up to 16 years compared to conventionally harvested SVGs (64 %) [28]. This effect can be attributed to the essential role of PVAT in vascular homeostasis [[29], [30], [31], [32]] and regulation of vascular tone [[33], [34], [35]]. PVAT is a heterogenous tissue mainly composed of pre-adipocytes, adipocytes, immune and nerve cells, as well as microvascular structures [36]. The properties of biomaterials, such as topography, stiffness and strength [24] are known to have significant effects on the innate immune response. It is therefore also possible that the material properties influence the formation of PVAT and its function.

We hypothesized that small-diameter vascular grafts (SDVGs) made of different materials with distinct biomechanical properties and structures would show differences already in the early immune response in anastomotic regions and PVAT formation. Since cytokine expression in response to biomaterials peaks during the 48 h of implantation [37] and the innate immune response resolves after approximately one week [38], these timepoints were chosen to observe processes that are significant in the early healing phase. Autologous, non-biodegradable ePTFE and biodegradable TPU/poly(urethane-urea) SDVGs (TPU/TPUU) were implanted as interposition grafts in the abdominal aorta of rats. Gene and protein expression analyses, cytokine expression arrays and immunohistochemistry were performed. In addition, the gene and protein expression of the newly formed PVATs, which were isolated after one week of implantation, were analyzed.

## 2 Materials and methods

### 2.1 Graft materials

For the *in vitro* studies, decellularized human umbilical cord arteries (dUCAs) were used as surrogates for autologous grafts in comparison to the synthetic grafts. The arteries were isolated from placentas from caesarean sections (week 37/0–40/0) obtained at the Department of Obstetrics and Gynecology at the Medical University of Vienna after informed consent of the mothers (EK: 1602/2018, Ethics Committee of the Medical University of Vienna). After isolation, the vessels were stored at −80 °C for 48 h. Decellularization was performed by consecutive washing steps under perfusion with phosphate buffer saline (PBS, Gibco, Germany), 1.4 % natrium chloride (NaCl, Sigma-Aldrich, Austria), distilled water, PBS and 24 h incubation with 1 % Triton X-100 (Sigma-Aldrich, Austria) with 0.02 % ethylenediaminetetraacetic acid (EDTA, Sigma-Aldrich, Austria). The arteries were perfused overnight with PBS, followed by several additional washing steps with PBS. Removal of residual DNA was performed by overnight incubation with 0.04 % DNAse I in PBS (including calcium and magnesium) at 4 °C in a petri dish. The DNAse solution was removed by washing the vessels with PBS for 30 min. The sterilization of the decellularized arteries was performed by incubation with 4.8 % ethanol +0.2 % peracetic acid (Sigma-Aldrich, Austria) for 1.5 h on a roller shaker. In a last step, the dUCAs were intensively rinsed with PBS three times and stored at 4 °C until use. Quantification of remaining DNA, collagen and glycosaminoglycan contents corresponded to the values of a previous study carried out by Schneider et al. (2020) [39]. TPU/TPUU was synthesized and electrospun as described before. The degradation of the designed degradable conduits results only in non-toxic aliphatic by-products. These grafts showed excellent biocompatibility and satisfying patency rates in long-term implantations [40]. The fabricated grafts had an inner diameter of 1.6 mm, similar to that of autologous rat aortas used for implantation. Aeos™ expanded polytetrafluoroethylene (ePTFE) grafts with an internodal distance of 30–100 μm, a wall thickness of 100 μm and an inner diameter of 1.5 mm were purchased from Zeus Industrial Products (USA).

### 2.2 Surface structure and biomechanics

The structure of the samples was studied using scanning electron microscopy (SEM). Graft specimens were dehydrated using an increasing ethanol series (25 %, 50 %, 60 %, 70 %, 80 %, 90 %, 96 % each for 10 min at room temperature). A final dehydration step with hexamethyldisilazane (HMDS, Sigma-Aldrich, Austria) was performed overnight. Dried samples were placed on holders, sputter coated with 40 nm gold and imaged with a Zeiss EVO 10 scanning electron microscope (Austria). By using the lookup table (LUT), histogram and surface plot tools in ImageJ (NIH, USA), the fiber distribution and surface structure was evaluated.

All mechanical experiments were performed in an isotonic salt solution at 37 °C. Tensile tests were performed using an ElectroForce LM1 testbench system (TA Instruments, USA) as published before [40,41]. Ring-shaped 2-mm graft segments were subjected to two aligned steel pins with a diameter of 0.6 mm. The pins were moved apart at a rate of 10 mm/min until failure of the specimens. Compliance was determined over a pressure range from 80 to 120 mmHg and expressed as the percent change in diameter per 100 mmHg.

### 2.3 Macrophage isolation and seeding

45 mL of whole blood were drawn from healthy adults by venipuncture after informed consent of the donors (Ethical approval: EK2321/2020, Ethics Committee of the Medical University of Vienna). The blood was collected in 3.2 % sodium citrate tubes (Greiner, Austria). 20 mL of blood were pipetted on top of 10 mL of Ficoll® Paque Plus (GE Healthcare, Austria) without mixing and centrifuged at 300×*g* for 30 min at room temperature without brake. The resulting peripheral blood mononuclear cell (PBMC) layer was transferred to a new tube, and washed twice with phosphate buffer saline (PBS, Gibco, Germany) with centrifugation for 10 min at 300×*g*. Cells were seeded to cell culture flasks in Roswell Park Memorial Institute (RPMI) 1640 medium (Sigma-Aldrich, Austria) supplemented with 10 % fetal bovine serum (FBS, Gibco, Germany). After 2 h of incubation, a medium change was performed and 50 ng/mL macrophage colony stimulating factor (M-CSF, R&D systems, USA) were added to stimulate macrophage differentiation. After three days, a medium change with addition of M-CSF was performed and macrophages were harvested for seeding onto graft materials on day 5 after isolation. 1 × 10^5^ macrophages were seeded onto dUCA, TPU/TPUU and ePTFE graft specimens with a size of 5 mm^2^ for 24 h and one week.

### 2.4 Immunocytochemistry

Immunofluorescence staining of macrophages seeded onto graft specimens *in vitro* was performed after one week of seeding time, similar to previous studies [[42], [43], [44]]. Samples were washed thrice with PBS and fixed in 4 % paraformaldehyde overnight at 4 °C. After fixation, samples were washed with PBS and subjected to PBS with 1 % bovine serum albumin (BSA, Sigma-Aldrich, Austria) for 1 h at room temperature. The samples were incubated for 1 h with the primary antibodies mouse anti-human CD68, rabbit anti-human CCR7 and rabbit anti-human CD163 (all from Abcam, USA) in a dilution of 1:100 in PBS/1 % BSA at room temperature. Specimens were washed thrice with PBS and incubated with 1:200 dilutions of secondary antibodies goat anti-mouse 647 and goat anti-rabbit 488 (both from Abcam, USA) for another hour. After staining, samples were again washed thrice with PBS, placed on microscopy slides, embedded in fluorescent mounting medium containing 4’,6-diamidine-2-phenylindole (DAPI, Thermo Scientific, Austria) and placed at 4 °C in the dark until imaging with a Zeiss LSM700 confocal microscope (Zeiss, Germany).

### 2.5 Real-time quantitative polymerase chain reaction (RT-qPCR)

RNA isolation for all RT-qPCR samples in this study was performed using a FavorPrep™ Tissue Total RNA purification kit (Favorgen, Austria) according to the manufacturer's instructions. The RNA yield and quality was assessed using a SPARK plate reader (TECAN, Austria). RNA with a 260/280 nm ratio higher than 2 was reversely transcribed to cDNA using a reverse transcription kit (Qiagen, Germany) and qPCR was performed using a CFX Opus 96 thermocycler (Biorad, Austria). Further information about the primer sequences is described in Supplementary Fig. S1.

### 2.6 Neutrophil attachment assay and neutrophil extracellular trap formation

Neutrophils were isolated from freshly donated human blood according to the protocol of Hsu et al. (2021) [45]. Briefly, 45 mL of blood was taken from human donors (ethical approval: EK2321/2020, Ethics Commission of the Medical University of Vienna) was collected in 3.2 % sodium citrate tubes (Greiner, Austria). 10 mL of blood was diluted with 35 mL of 5 % fetal bovine serum (FBS)/Hank's Balanced Salt Solution (HBSS) and the tube was inverted to mix. The diluted blood was carefully pipetted onto Ficoll® Paque Plus and centrifuged at 400×*g* for 30 min at room temperature. The PBMC layer was then removed and the erythrocyte/neutrophil pellet was transferred to a new tube and resuspended in 5 % FBS/HBSS to a final volume of 25 mL. 25 mL of prewarmed 3 % dextran (Sigma-Aldrich, Austria) in 0.9 % NaCl/H_2_O was added and the suspension was incubated for 15 min on a non-vibrating surface. Subsequently, the upper layer was collected and centrifuged at 400×*g* with low brake for 10 min at room temperature. The supernatant was removed from the resulting red pellet and the cells were gently resuspended with sterile water for 28 s to induce lysis of the red blood cells. Immediately, 1.8 % NaCl/H_2_O was gently added. The solution was centrifuged at 200×*g* with the brake off for 5 min. Neutrophils were resuspended in 10 % FBS/RPMI 1640 (Sigma-Aldrich, Austria), counted, and adjusted to 5 × 10^6^ cells/100 μL. 1 × 10^6^ neutrophils were seeded on graft samples (2 × 2 mm) for 1 h at 37 °C. Unattached neutrophils were removed by three gentle washes with PBS. The cells on the various substrates were detached and counted using a hemocytometer.

The neutrophils used for neutrophil extracellular trap (NET) formation were isolated and seeded onto the materials as described above. Scanning electron microscopy (SEM), DAPI staining and immunofluorescence labelling against myeloperoxidase (MPO) were performed to visualize and quantify the number of NETs on the different materials. Quantification of NETs was performed similarly to Abaricia et al. (2021) [46].

In addition to the neutrophil attachment assay, neutrophils seeded to graft specimens to evaluate NET formation were visualized with SEM. Glass coverslips (VWR, Germany) were used as control. For this purpose, the graft specimens were washed thrice with PBS after the 1-h neutrophil attachment. 2.5 % glutaraldehyde was added overnight at 4 °C. Samples were again washed three times with PBS and further prepared for SEM as described above.

### 2.7 Graft implantations and sample collection

Autologous, TPU/TPUU and ePTFE grafts were implanted into the infrarenal abdominal aorta of male Sprague Dawley rats weighing between 450 and 600 g (Supplementary Fig. S2). The study was approved by the Institutional Ethics Committee and the Federal Ministry of Science and Research (BMWFW-66.009/368-V/3b/2019). All animals received human care in accordance with the Good Scientific Practice guidelines of the Medical University of Vienna. Animals were anesthetized intraperitoneally with 100 mg/kg ketamine and 5 mg/kg xylazine. Prior to surgery, the rats received 30 mg/kg piritramide and 7.5 mg/kg enrofloxacin. During surgery, rats were ventilated with 40 % oxygen and 2 % isoflurane. The infrarenal aorta was dissected from surrounding tissue and the vena cava. Afterwards, the aorta was clamped and approximately 1 cm was removed. Autologous grafts were flushed with heparinized saline and immediately transplanted. TPU/TPUU and ePTFE grafts were stored in water supplemented with 5 IU/mL heparin 1 h prior to implantation. The implantation was performed by single knot technique using 10/0 nylon (Monosof™, Covidien, Austria). As perioperative analgetic therapy 3 mg/kg piritramide (Janssen-Cilag Pharma, Austria) and 5 mg carprofen (Rimadyl®, Pfizer, Austria) were used. Later piritramide was given through the drinking water during the first days after surgery. The efficiency of the analgetic regimen was individually evaluated and adapted according to the rat grimace scale. The rats did not receive any anti-coagulation therapy during or after surgery.

Grafts were retrieved after 24 h and one week. Anesthesia and pain relief were administered in a similar manner to the implantation procedure. Immediately prior to implant removal, the animals were given 100IU heparin (Gilvasan, Austria) to prevent thrombus formation due to blood stasis caused by clamping of the host vessel. The anastomoses were subjected to PBS, immediately homogenized and frozen at −80 °C in PBS with 1 % Triton-X for at least 24 h. Samples for immunohistochemistry were fixed in 4 % PFA. 2 mm sections of each graft were tap-frozen in liquid nitrogen for protein isolation and sodium dodecyl sulfate polyacrylamide gel electrophoresis (SDS-PAGE). Graft specimens for RT-qPCR were subjected to RNAlater buffer (Thermo Scientific, Austria) and frozen at −80 °C until RNA isolation. From one-week implantations, PVAT was isolated from the graft adventitia. Part of the PVAT was used for outgrowth cultures, and the remaining PVAT was used for RT-qPCR.

### 2.8 SDS-PAGE

Two samples of TPU/TPUU and ePTFE were taken to determine the adsorption of proteins after 24 h of implantation. The shock-frozen samples were ground with a glass mortar in 100 μL protein lysis buffer (200 mM 2-amino-2-(hydroxymethyl)-1,3-propandiol (TRIZMA base), 1.37 M NaCl and 100 mM EDTA in glycerol, all from Sigma-Aldrich (Austria)). The ground samples were incubated on ice for 30 min and then centrifuged at 4 °C for 40 min at 18,000×*g*. The protein content of the lysates was measured using a Modified Lowry Protein Assay Kit (Thermo Scientific, Austria). 20 μg of protein was loaded per lane of a 10 % acrylamide gel. The gel was run at 40 V for 5 h in a Bio-Rad gel chamber (Bio-Rad, Austria) and stained with a Coomassie blue staining solution (Imperial Protein Stain, Thermo Scientific, Austria). The de-staining was carried out with a self-made de-stain solution (10 % glacial acetic acid and 20 % methanol in sterile water) eight times for 15 min each at room temperature.

### 2.9 Immunohistochemistry

PFA-fixed samples were embedded in optimal cutting temperature (OCT) medium (Fisher Scientific, Austria) and cryo-sectioned to 5 μm slices using a CryoStar NX70 cryotome (Thermo Scientific, Austria). Sections were frozen at −20 °C overnight. OCT was removed from sections using ice cold acetone for 4 min and a subsequent decreasing ethanol series (90 %, 80 %, 70 %, 60 % each for 4 min at room temperature). The staining procedure for tissue slices was identical to immunocytochemistry staining of *in vitro* samples. Quantification was performed by using the cell counting plugin of ImageJ (NIH, USA).

### 2.10 Cytokine array

Anastomosis samples frozen for cytokine arrays were processed using Proteome Profiler Rat XL Cytokine Array kits (R&D systems, Germany) according to manufacturer's instructions. Briefly, protein lysates of anastomosis samples were incubated on array membranes overnight. Subsequently, primary and secondary antibody cocktails were added and signals were obtained using a ChemiDoc imaging system (Bio-Rad, Austria). Clustering was performed using the ClusterMaker2 plugin in Cytoscape [47], which offers several clustering algorithms to identify densely connected groups within the network. After applying ClusterMaker2, the resulting clusters were ranked by size, from the largest to the smallest, and unconnected nodes were listed at the end. Only clusters containing more than three nodes were included. The STRING app in Cytoscape [48] was used to construct the protein-protein interaction network. The "interaction confidence threshold" in STRING defines the minimum confidence score for including an edge between two proteins, based on integrated data sources such as curated pathways, text mining, co-expression, experimental data, and known complexes. To determine the optimal threshold, networks were initially generated using STRING's default threshold of 0.5, as well as higher thresholds of 0.7 and 0.9. While lower thresholds produced denser networks with larger subclusters, the high-confidence threshold of 0.9 for presentation to focus on the most reliable associations was selected. This decision prioritizes biological relevance and minimizes potential false positives.

### 2.11 PVAT outgrowth cultures

Isolated approximately 1 cm^3^ large PVAT tissue samples were placed in 6 well plates scratched with a scalpel blade to facilitate cell attachment. 2 mL of endothelial growth medium-2 (EGM-2, Lonza, Switzerland) supplemented with 10 % of FBS (Gibco, Germany) and 1 % penicillin/streptomycin were added. Outgrowth cultures were incubated in cell culture environment until outgrown cells showed 40 % confluence. Cells were detached using trypsin-EDTA (Gibco, Germany) and transferred to cell culture flasks. Medium change was performed every second day and cells were passaged at 80 % confluence.

### 2.12 PVAT characterization

Cells from PVAT outgrowth cultures were characterized using immunofluorescence staining and gene expression analyses. Staining was performed according to the protocol described for immunocytochemistry above. Mouse anti-rat CCR7, rabbit anti-S100A4, rabbit anti-adiponectin, mouse anti-rat CD73, and secondary antibodies goat anti-mouse 647 and goat anti-rabbit 488 were purchased from Abcam (USA). Mouse anti-rat CD163 antibody was acquired from Santa Cruz Biotechnology (USA). Rabbit anti-rat CD105 antibody was purchased from Bioss (USA). Fluorescence signals were quantified with ImageJ (National Institute of Health, USA) from five images of three individual rats per condition. Gene expression analyses were performed using RT-qPCR as described above.

### 2.13 Statistical analysis

Data presentation and statistical calculations were performed using Prism 10.0 software (GraphPad, USA). *In vitro* experiments were performed in biological replicates of at least n = 6 if not otherwise stated. For *in vivo* analyses, samples from five individual animals were taken from each condition (autologous, ePTFE, TPU/TPUU). All experiments were performed in technical triplicates and significant differences were calculated using a one-way analysis of variance (ANOVA) and Tukey post-test. Normal distribution of measurements was evaluated using Shapiro-Wilk normality testing. In case of non-normal distribution, non-parametric tests were performed. Significance levels were indicated by asterisks (∗ = p ≤ 0.05, ∗∗ = p ≤ 0.01, ∗∗∗ = p ≤ 0.001).

## 3 Results

### 3.1 Material characteristics

**Fig. 1:**
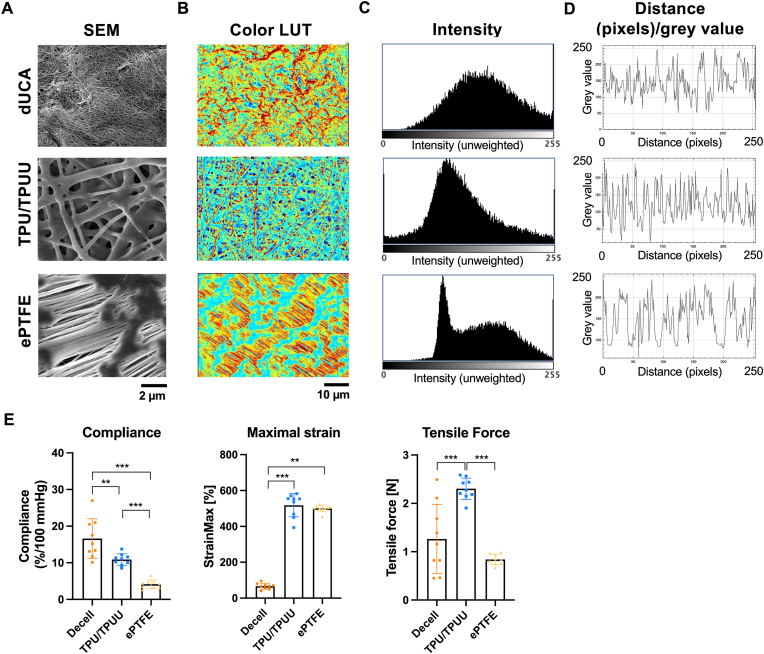
**Structural and biomechanical evaluation of graft materials.** dUCA grafts showed a smooth and uniform fiber distribution, TPU/TPUU had a porous structure with a relatively uniform fiber size and distribution and ePTFE showed distinct structural differences between nods and rods with a rough surface (A–D). Biomechanical evaluation showed that dUCA grafts had the highest compliance whereas TPU/TPUU withstood the highest tensile force and both synthetic grafts showed an increased maximal strain (E).

The characteristics of the three graft materials were compared in terms of morphology and biomechanics. Fig. 1 shows the structural differences between native dUCA, TPU/TPUU, and ePTFE grafts. Intensity analyses of SEM images showed a more uniform surface with thin fibers and small pores on the surface of dUCA compared to the synthetic graft materials (Fig. 1A–D). Decellularized UCA had a significantly higher compliance than TPU/TPUU and ePTFE. TPU/TPUU showed a higher tensile strength than dUCA and ePTFE. Both synthetic materials showed a significantly increased maximal strain. (n = 9, Fig. 1E).

### 3.2 Decellularized UCA and TPU/TPUU grafts induced anti-inflammatory protein and gene expression of macrophages

**Fig. 2:**
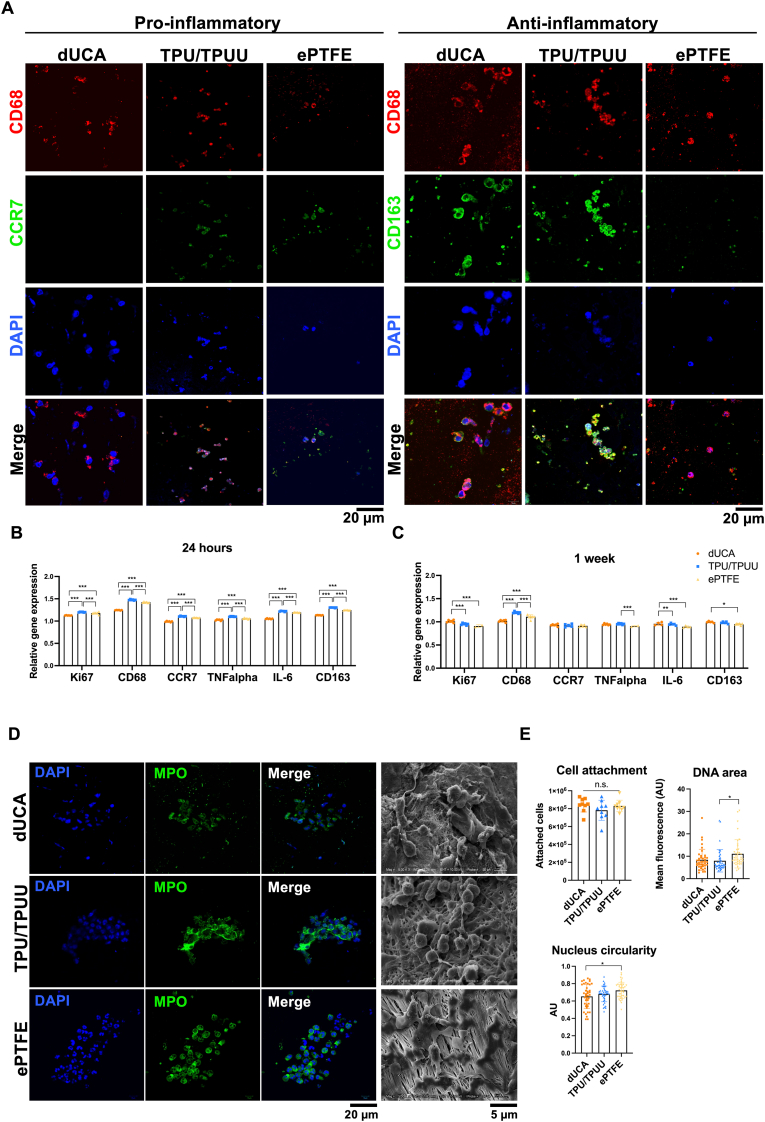
**Immune cell activation.** Macrophages seeded onto TPU/TPUU and ePTFE grafts *in vitro* expressed low levels of CCR-7 whereas CD163 expression was absent on ePTFE (A). Gene expression analyses showed a significantly increased expression of pro-inflammatory cytokines after 24 h of seeding (B). After one week, ePTFE showed the lowest cytokine as well as Ki67 expression, indicating reduced cell viability (C). In addition to SEM imaging, a MPO staining to evaluate NET formation was performed (D). The number of attached neutrophils was similar between the conditions, whereas ePTFE showed the highest DNA area. The MPO expression showed a trend towards higher expression on TPU/TPUU (E).

To evaluate the materials' potential to trigger a pro-inflammatory response in macrophages, the cells were seeded on dUCA, TPU/TPUU and ePTFE samples for one week. Immunofluorescence staining showed a slightly increased expression of the pro-inflammatory CCR7 on TPU/TPUU and ePTFE. However, CD163 was significantly increased on dUCA and TPU/TPUU samples, thus indicating a differentiation of macrophages towards the anti-inflammatory M2 phenotype. Furthermore, a reduced cell and nucleus size was detected in macrophages seeded on TPU/TPUU and ePTFE (Fig. 2A). Gene expression was evaluated after 24 h and one week of seeding (n = 6). After 24 h (Fig. 2B), the synthetic graft materials showed significantly increased expression of proliferation-associated Ki67, pan-macrophage marker CD68, pro-inflammatory genes CCR7 and IL-6, and anti-inflammatory genes. Interestingly, macrophages on synthetic materials showed significantly reduced proliferation (Ki67) after one week. Likewise, the expression of TNFα, IL-6 and CD163 was significantly reduced. CCR7 values remained unchanged compared to 24 h. CD68 gene expression remained significantly higher after one week in cells seeded on TPU/TPUU and ePTFE (Fig. 2C).

### 3.3 No material-related influence on NET formation *in vitro*

Fig. 2D and E shows the adhesion and NET formation of human neutrophils on the various vascular graft materials (n = 4). Quantification of neutrophil adhesion on different vascular graft materials showed no significant effect of the substrate on adhesion. However, TPU/TPUU showed a slight trend towards reduced neutrophil adherence. To evaluate neutrophil activation, NET formation was examined by DAPI and MPO staining. Quantification of the DNA area (DAPI staining) showed a significantly higher nuclear area on ePTFE compared to dUCA and TPU/TPUU. A trend towards higher MPO expression was observed for TPU/TPUU, but the difference was not significant.

### 3.4 Similar protein adsorption on TPU/TPUU and ePTFE grafts

**Fig. 3:**
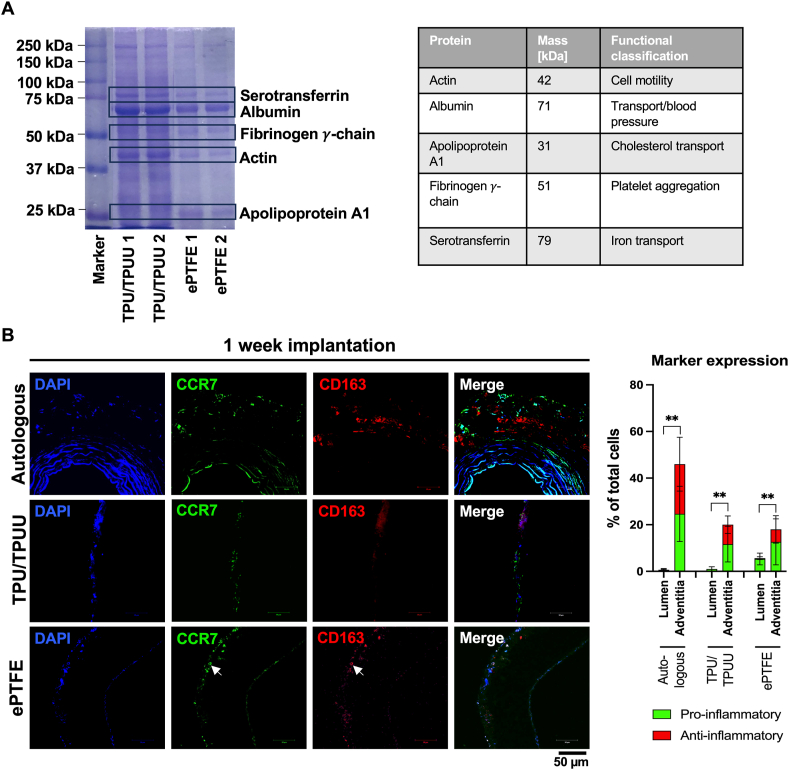
**Protein adsorption and immune cell infiltration after one week of implantation**. A similar adsorption of proteins mainly involved in the coagulation cascade and fatty acid transport was found on TPU/TPUU and ePTFE (A). Autologous and ePTFE grafts both showed the attachment and infiltration of pro-inflammatory CCR7^+^ and anti-inflammatory CD163^+^ macrophages, whereas significantly more cells were located in the lumen than adventitia (B).

After 24 h of implantation, the protein abundance on the luminal layer of the synthetic graft materials was evaluated using SDS-PAGE (n = 2). Similar to the findings of Fröhlich et al. (2015), the proteins actin, albumin, apolipoprotein A1, the fibrinogen γ-chain and serotransferrin were adsorbed [49] (Fig. 3A).

### 3.5 ePTFE grafts promoted recruitment of CCR7^+^ macrophages to the lumen

Fig. 3B shows immunohistochemistry staining of vascular grafts that were implanted in rats for one week. The adventitial tissue of autologous implants exhibited CCR7^+^ cells located mostly in the border area. CD163 showed only limited specific staining in the adventitial area. TPU/TPUU grafts exhibited CCR7^+^ cells on the graft adventitia and only few CD163^+^ cells in the whole graft area. Interestingly, CCR7^+^ cells attached to the adventitia as well as to the luminal side of ePTFE grafts. Furthermore, CD163^+^ cells were present on the adventitia. The staining indicated that some cells co-expressed both proteins (indicated by arrows). Quantification of the cell populations showed that under all three conditions, significantly more macrophages were found on the adventitial side than in the lumen. While the number of pro- and anti-inflammatory macrophages in the adventitia was balanced in autologous and TPU/TPUU implants, ePTFE grafts showed an increased number of pro-inflammatory macrophages.

### 3.6 Increased inflammation in anastomotic regions of synthetic grafts

**Fig. 4:**
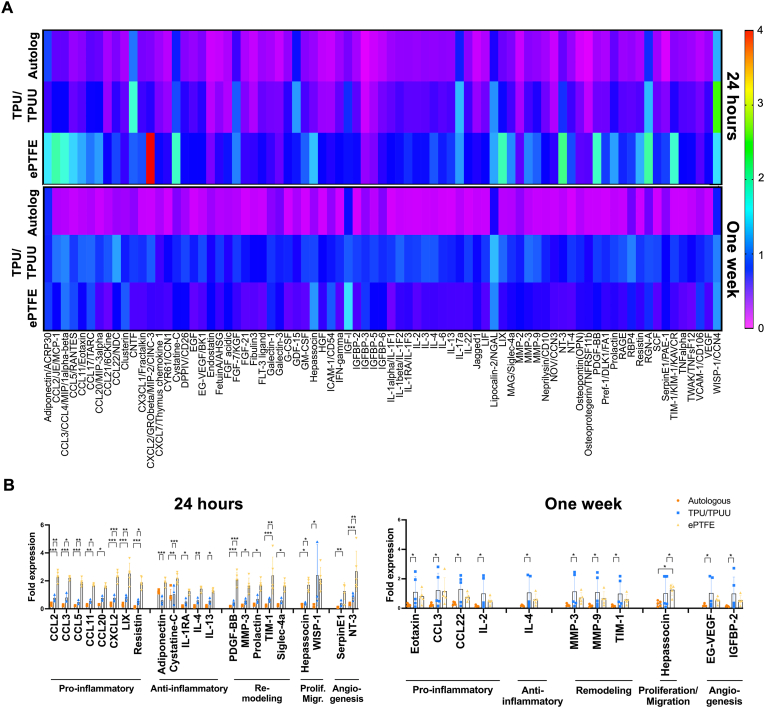
**Cytokine arrays of anastomotic regions.** Autologous grafts showed a significant lower cytokine expression in the anastomoses compared to TPU/TPUU and ePTFE. The highest cytokine expression was observed in anastomotic regions of ePTFE grafts after 24 h of implantation (A–B).

Cytokine arrays showed that implantation of ePTFE grafts induced significantly higher expression of the tested cytokines in the anastomosis areas after 24 h (n = 5, Fig. 4A). In particular, the chemokine ligand CXCL2 was expressed approximately four times higher compared to autologous and TPU/TPUU implants. Interestingly, inflammation in ePTFE anastomosis regions decreased after one week, whereas biodegradable TPU/TPUU showed a delayed inflammatory response after one week. Cytokine expression levels in the autologous anastomoses remained low and were largely unchanged from 24 h to one week. Table 1 and Fig. 4B summarize the expression levels and function of all cytokines that were significantly differentially expressed between the conditions (mean ± SD).

**Table 1:** Cytokine expression in anastomotic regions.

*24 h*		*Autologous*	*TPU/TPUU*	*ePTFE*	*Function*
Pro-inflammatory	CCL2	0.497 ± 0.332	0.775 ± 0.229	1.870 ± 1.037	Immune cell activation, leukocyte infiltration, T-cell proliferation [50]
	CCL3	0.494 ± 0.412	0.799 ± 0.328	1.714 ± 0.888	Differentiation of IL-2 expressing Th-1 T-cells [51]
	CCL5	0.328 ± 0.196	0.476 ± 0.154	1.470 ± 0.815	Expressed in ischemia-reperfusion injury [52]
	CCL11	0.373 ± 0.198	0.549 ± 0.126	1.336 ± 0.692	Induced by high shear stress, mediates smooth muscle cell migration [53,54]
	CCL20	0.471 ± 0.297	0.773 ± 0.168	1.293 ± 0.651	Sepsis [55], inflammatory diseases [56,57]
	CXCL2	0.400 ± 0.371	0.676 ± 0.304	4.003 ± 2.400	Giant cell chemoattractant and neutrophil adhesion [58]
	LIX	0.460 ± 0.335	0.836 ± 0.174	1.829 ± 1.197	Expressed in ischemia-reperfusion injury [52]
	Resistin	0.268 ± 0.262	0.552 ± 0.143	1.356 ± 0.857	Atherosclerosis [59,60]
Anti-inflammatory	Adiponectin	1.014 ± 0.431	0.603 ± 0.208	1.534 ± 0.653	Suppression of SMC proliferation [61]
	Cystatine-C	0.881 ± 0.52	1.029 ± 0.709	1.780 ± 0.624	Acts anti-inflammatory in atherosclerosis [62]
	IL-1RA	0.342 ± 0.271	0.510 ± 0.247	0.932 ± 0.512	Suppressor of intimal hyperplasia [63]
	IL-4	0.347 ± 0.295	0.593 ± 0.205	1.064 ± 0.596	Mediates Th2 differentiation of T-cells [64], inhibits inflammation [65]
	IL-13	0.351 ± 0.276	0.571 ± 0.202	0.972 ± 0.506	Tissue repair and suppression of inflammation [65]
Remodeling	PDGF-BB	0.293 ± 0.196	0.454 ± 0.150	1.77 ± 1.098	Overexpression increases CCL-2 in vascular grafts [66], SMC proliferation [61]
	MMP-3	0.371 ± 0.242	0.587 ± 0.168	1.221 ± 0.774	Overexpression inhibits SMC migration [67]
	Prolactin	0.489 ± 0.329	0.700 ± 0.293	1.301 ± 0.676	Protective role in trauma-induced hemorrhage [68], protects against inflammation in severe stress conditions [69]
	TIM-1	0.372 ± 0.264	0.694 ± 0.135	1.749 ± 1.399	B_reg_ marker, prevention of organ transplant rejection [70]
	MAG	0.404 ± 0.254	0.587 ± 0.205	1.233 ± 0.696	Nerve fiber formation [71]
Proliferation/Migration	Hepassocin	0.322 ± 0.135	0.447 ± 0.076	1.410 ± 0.727	Acute inflammation [72]
	WISP-1	1.344 ± 2.123	2.398 ± 2.192	1.507 ± 1.119	Tissue remodeling [73]
Angiogenesis	SerpinE1	0.119 ± 0.053	0.387 ± 0.224	0.999 ± 0.748	Pro-angiogenic [74]
	NT-3	0.450 ± 0.289	0.944 ± 0.229	1.969 ± 1.524	Mobilization and homing of endothelial progenitor cells [75,76]

**One week**		*Autologous*	*TPU/TPUU*	*ePTFE*	*Function*

Pro-inflammatory	CCL11	0.224 ± 0.086	1.105 ± 1.049	0.816 ± 0.523	Induced by high shear stress, mediates smooth muscle cell migration [53,54]
	CCL3	0.231 ± 0.098	1.215 ± 1.168	1.155 ± 1.054	Differentiation of IL-2 expressing Th-1 T-cells [51]
	CCL22	0.314 ± 0.101	1.302 ± 0.946	0.777 ± 0.316	Induced by IL-4 expression [77], also expressed upon contact with microbial products
	IL-2	0.154 ± 0.054	0.999 ± 1.041	0.523 ± 0.302	Expressed by Th-1 T-cells [51], high levels associated with acute transplant rejection [78]
Anti-inflammatory	IL-4	0.177 ± 0.039	1.065 ± 1.064	0.609 ± 0.428	Mediates Th2 differentiation of T-cells [64], inhibits inflammation [65]
Remodeling	MMP-3	0.219 ± 0.077	1.138 ± 1.127	0.726 ± 0.408	Overexpression inhibits SMC migration [67]
	MMP-9	0.178 ± 0.074	1.087 ± 1.076	0.671 ± 0.526	Facilitates immune cell migration [79]
	TIM-1	0.166 ± 0.054	0.987 ± 0.953	0.602 ± 0.389	B_reg_ marker, prevention of organ transplant rejection [70]
Proliferation/Migration	Hepassocin	0.316 ± 0.181	1.013 ± 1.060	1.266 ± 0.444	Acute inflammation [72]
Angiogenesis	EG-VEGF	0.200 ± 0.082	1.034 ± 1.006	0.558 ± 0.260	Produced by endocrine glands, pro-angiogenic [80,81]
	IGFBP-2	0.135 ± 0.034	0.994 ± 1.126	0.554 ± 0.369	Enhances VEGF promotor activity

The cytokine arrays were subjected to gene ontology enrichment to group the observed cytokines according to molecular function and biological processes (Supplementary Fig. S3). Most of the observed genes encoding the measured proteins play a role in cytokine and receptor ligand activity, as well as in signal receptor binding and the regulation of molecular functions. The most common processes affected by the cytokines were (cellular) responses to IL-1, neutrophil chemotaxis, and granulocyte and myeloid leukocyte migration.

**Fig. 5:**
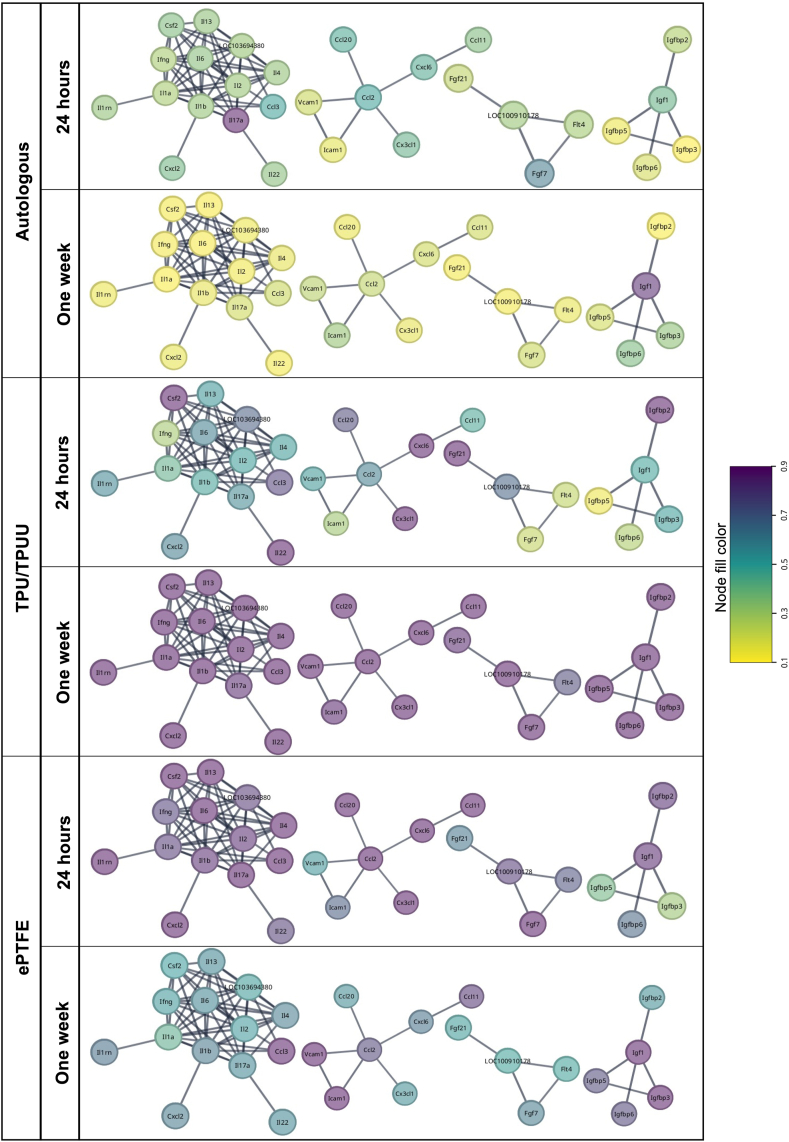
**Gene clusters of regulated cytokines.** Gene clusters of analyzed cytokines in the anastomotic regions showed that cytokine levels in autologous regions remained low between 24 h and one week. Interestingly, the expression of cytokines increased in TPU/TPUU anastomoses from 24 h to one week whereas the expression decreased in ePTFE implants, thus indicating a delayed inflammatory response in TPU/TPUU vascular grafts.

The protein interaction networks shown in Fig. 5 revealed various clusters and interactions between proteins that are important for understanding biological processes related to inflammation and host response, particularly in the context of interactions between tissue materials. After 24 h, ePTFE samples in particular showed strong activation of a network containing various interleukins and another network containing the chemokine ligands CCL2, CCL20, CCL11, CXCL6, and CX3CL1. When considering the same networks of the other conditions, only individual proteins were expressed in TPU/TPUU (IL-22, Csf2, CX3CL1, CXCL6) and autologous (IL-17a) samples. Both synthetic materials showed an upregulation of IGFBP-2, with ePTFE also expressing the interaction partner IGF-1. In addition, ePTFE samples showed a strong involvement of the fibroblast growth factor (FGF) family, while TPU/TPUU anastomoses only expressed FGF21. No upregulation of FGFs was observed in the autologous samples. After one week, only IGF-1 and CCN4 were expressed in autologous anastomoses. Interestingly, TPU/TPUU showed significant activation of all networks involved. The strong activation of the interleukin network in ePTFE samples was reduced after one week. CCL3 remained the only highly expressed protein after one week. While ICAM-1 and VCAM-1 were weakly expressed in ePTFE anastomoses after 24 h, both proteins were upregulated after one week. A further change was observed in the IGFBP family network. IGF-1 remained highly expressed, but instead of its interaction partner IGFBP-2, other network members, IGFBP-3, IGFBP-5 and IGFBP-6, were activated.

### 3.7 PVAT from ePTFE implants showed increased inflammation and decreased regenerative capacity

**Fig. 6:**
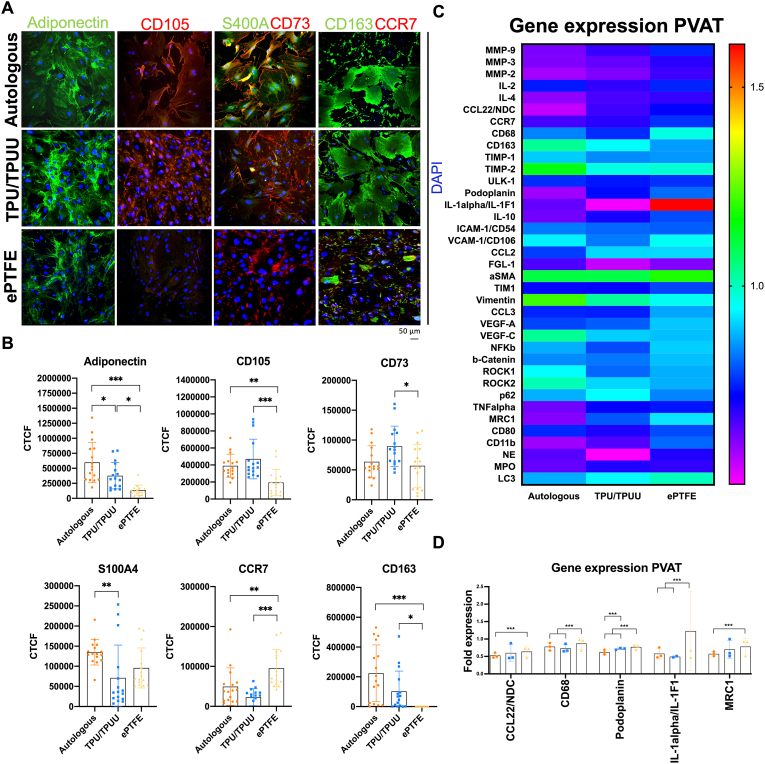
**Evaluation of PVAT composition.** Outgrowth cultures from PVATs derived from the different implanted graft materials after one week showed a significantly higher expression of pro-inflammatory markers as well as a reduction of regenerative and anti-inflammatory proteins in ePTFE PVATs (A,B). Similarly, pro-inflammatory gene expression was enhanced in ePTFE PVATs where IL-1alpha showed the highest upregulation (C,D).

Outgrowth cultures of one-week PVATs were subjected to immunofluorescence staining for various regenerative and inflammation-associated markers (Fig. 6A). Quantification of the fluorescence signals (n = 5, Fig. 6B) displayed as corrected total cell fluorescence (CTCF) revealed a significantly higher expression of adiponectin in PVATs of autologous SDVGs compared to TPU/TPUU and ePTFE. Regenerative/stem cell markers CD105 and CD73 were significantly down-regulated in PVATs of ePTFE. The calcium-binding protein S100A4 was significantly lower expressed in PVATs of TPU/TPUU compared to autologous PVATs. Whereas the pro-inflammatory CCR7 was significantly increased in PVATs of ePTFE compared to autologous and TPU/TPUU, the anti-inflammatory marker CD163 was significantly reduced in ePTFE-derived PVATs.

### 3.8 Increased pro-inflammatory gene expression in PVAT of ePTFE implants

Gene expression analyses of PVATs retrieved from one-week implants were analyzed for genes associated with healing and inflammation (n = 5, Fig. 6C). Quantification of the expression profiles revealed five genes that were significantly different expressed between the conditions (Fig. 6D). Similar to the observations for cytokine protein expression in the anastomotic regions, the CCL22 gene was higher expressed in PVATs of ePTFE implants. Furthermore, the presence of macrophages indicated by CD68 expression was increased in ePTFE samples compared to autologous and TPU/TPUU PVATs. The lymphatic marker podoplanin was higher expressed in both synthetic graft conditions compared to autologous grafts. Pro-inflammatory IL-1α showed a considerably higher expression in ePTFE PVATs. Additionally, mannose receptor C-type 1 (MRC1) was significantly increased in PVATs of ePTFE implants.

## 4 Discussion

For over 70 years, autologous vessels have remained the gold standard for small blood vessel replacement [82,83]. Ideally, the healing characteristics of synthetic conduits should be similar to those of autologous grafts to improve their performance. The present study aimed to investigate differences and similarities of the early inflammatory response and healing processes in autologous grafts compared to ePTFE and biodegradable TPU/TPUU SDVGs. In particular, cytokine expression in anastomotic regions and PVAT inflammation were observed in detail. *In vitro* and *in vivo* experiments revealed differences in the intensity of inflammatory activation and the timing of inflammatory responses.

The initial immune response to biomaterials includes the adsorption of proteins with subsequent recruitment and attachment of neutrophils and macrophages [38]. It has already been shown that the surface properties and chemical composition of biomaterials can strongly influence the protein adsorption [84] as well as viability, adhesion and activation of immune cells [24]. DUCAs consist mainly of cytoskeletal proteins, proteoglycans, collagen and other extracellular matrix proteins [85]. In previous work, the decellularization procedure was optimized to ensure DNA removal and excellent biocompatibility of the conduits [86]. These grafts showed a significant reduction in pro-inflammatory gene expression seven days after implantation and induced a significant phenotype switch from pro-inflammatory to anti-inflammatory macrophages after 21 days [85]. Other studies using dUCAs reported limited inflammation in the early phase and the absence of chronic inflammatory processes in long-term implantations [87,88]. TPU/TPUU grafts were fabricated from a blend of biodegradable polyester urethane and polyester urethane-urea. In an earlier study, no significant changes in pro- or anti-inflammatory gene expression were observed between short-term (one week) and long-term (three and six months) implantation times for TPU/TPUU. In general, these grafts showed excellent biocompatibility, and no toxic degradation products were found in the parenchymal organs at any time point of implantation [40]. As TPU/TPUU is a biodegradable material, the inflammatory response observed in the macrophage seeding experiment could, as previously reported, be due to graft degradation [[19], [20], [21]]. ePTFE is a hydrophobic material that induced a significantly prolonged pro-inflammatory immune response compared to polyurethanes *in vitro* and *in vivo* [42,43].

DUCA had a smoother and more uniform surface than TPU/TPUU and ePTFE grafts. The evaluation of biomechanical properties showed that dUCA and TPU/TPUU materials had similar compliance values to coronary arteries (8–17 %/100 mmHg) [89], whereas ePTFE had a significantly lower compliance. The tensile force was highest for TPU/TPUU and was in the range of native arteries [[89], [90], [91], [92]]. Wang et al. (2024) compared the non-linear elasticity of synthetic SDVGs with that of native vessels, demonstrating that there is still a biomechanical mismatch that can lead to reduced graft patency [93]. Furdella et al. (2021) showed that less compliant vascular grafts mediated an increased pro-inflammatory immune response compared to compliant constructs [94]. Further investigations indicated that non-physiological cyclic strain values of polymeric scaffolds led to enhanced inflammation and especially an increased M1/M2 macrophage ratio [95]. Both, TPU/TPUU and ePTFE showed significantly higher strain values and triggered stronger pro-inflammatory reactions than dUCAs, suggesting that tensile strain may also influence macrophage activation. In particular, ePTFE induced a higher protein expression of pro-inflammatory CCR7 and reduced levels of anti-inflammatory CD163. A stronger anti-inflammatory phenotype of macrophages seeded on electrospun fibers compared to flat substrates was observed in previous studies [[96], [97], [98], [99]]. A study using polydioxanone scaffolds indicated an enhanced anti-inflammatory macrophage phenotype with increasing fiber diameter and higher porosity [97]. The fiber diameter of these scaffolds correlated with that of the TPU/TPUU used in the current study and ranged from 2 to 6 μm [40], and indeed macrophages seeded on TPU/TPUU showed increased expression of anti-inflammatory CD163 compared to ePTFE. Schutte et al. (2008) reported that macrophage polarization on biocompatible polymers was generally low [100], while Bhardwaj et al. (2001) actually showed that Teflon induced higher expression of pro-inflammatory TNFα as well as decreased macrophage viability after 10 days of *in vitro* culture [101]. In the current study, ePTFE and TPU/TPUU induced higher gene expression of the pro-inflammatory markers CCR7, TNFα and IL-6 after 24 h of macrophage seeding compared to the decellularized artery. This goes in line with other studies where dUCAs showed a limited pro-inflammatory immune response [102,103]. Interestingly, the expression of all markers decreased in macrophages seeded on ePTFE after one week. This phenomenon could be related to the decreased Ki67 levels, which indicate that the macrophages are no longer viable and undergo apoptosis. Immunostaining showed that macrophages seeded on synthetic TPU/TPUU and ePTFE had a reduced nucleus and cell size compared to macrophages seeded on dUCAs. Possibly, cells seeded on synthetic materials remained in the G0/G1 phase of the cell cycle, whereas macrophages on dUCA entered the G2 phase, which is characterized by a significant increase in nucleus and cell volume [104]. Porcheray et al. (2005) showed that there is no clear correlation between macrophage morphology and pro- or anti-inflammatory stimuli [105]. However, another study showed that pro-inflammatory stimulation of macrophages with TNFα led to membrane disintegration and significant changes in cell morphology towards a more expanded phenotype but no changes in nucleus size were observed [106]. Therefore, we concluded that the changes in nucleus and cell size were due to changes in the cell cycle rather than an inflammatory response.

Chang et al. (2003) showed that ePTFE and Dacron induced rapid neutrophil apoptosis due to the high surface roughness of the materials [107]. In the present study, the number of adhered neutrophils and NET formation was similar between the native and synthetic materials. As NETs can cause uncontrolled inflammation, pro-inflammatory activation of neutrophils and tissue damage [108], the absence of excessive NET formation underlines the biocompatibility of all materials used.

After the evaluation of the inflammatory potential of the different materials *in vitro*, implantations with autologous, TPU/TPUU and ePTFE vascular grafts were performed for 24 h and one week in rats. Within milliseconds after biomaterial implantation, proteins start to adhere to the material surface to facilitate subsequent immune cell attachment [84,109]. These proteins are mainly associated with the coagulation cascade and complement system and their adhesion is highly dependent on the surface structure, charge and wettability of the biomaterial [84,109]. Similar to the results obtained by Fröhlich et al. (2015), no significant differences in the protein content of polyurethane and ePTFE grafts were observed in the present study-despite varying surface characteristics. The adsorbed proteins are mostly participating in lipid [110,111] or iron transport [112] and the coagulation cascade [113].

Since pro-inflammatory macrophage infiltration and a switch towards the M2 phenotype is a key determinant for successful vascular graft healing [114], the presence of pro- and anti-inflammatory macrophages in the graft wall after one week of implantation was assessed. Autologous, TPU/TPUU and ePTFE grafts showed both pro-inflammatory CCR7^+^ and anti-inflammatory CD163^+^ cell populations in the graft wall, indicating an ongoing phenotype switch from M1 to M2. CCR7 has been shown to be important in inducing the recruitment of T lymphocytes from peripheral tissues to specific sites such as the blood vessel wall [115]. The presence of CCR7^+^ cells on the adventitial side of the graft material could therefore be a first step towards the involvement of the adaptive immune system through the recruitment of T cells. CD163^+^ macrophages were abundant in the wall of autologous grafts, which goes in line with the findings of Rubio-Navarro et al. (2015) who reported low expression of CD163 in healthy human aortas [116]. Few CD163^+^ macrophages were found on the adventitial side of ePTFE grafts. Interestingly, TPU/TPUU graft lumen remained free of CD163^+^ macrophage adhesions, indicating a delay of pro-healing macrophage polarization in these grafts compared to ePTFE.

Anastomotic regions of synthetic implants are prone to intimal hyperplasia formation due to differing biomechanical properties of the graft material and the native vessel [117]. In the current study, a cytokine array was performed with samples from anastomoses to analyze 79 different cytokines that mediate pro- and anti-inflammatory processes, remodeling, cell proliferation and migration, and angiogenesis. The data were normalized to expression levels of native, unoperated arteries. Autologous grafts did not show any cytokine upregulation, which can be attributed to the matched compliance and genetic identity with the host aorta since the grafts were transplanted into the same rat. In contrast, ePTFE showed particularly high pro-inflammatory cytokine expression after 24 h, whereas TPU/TPUU showed higher cytokine levels after one week. It is likely that the decrease in inflammation with ePTFE is due to the fact that immune cells were unable to interact with the material and consequently no further immune response signaling cascade was triggered [118]. The inflammatory response to biodegradable materials differs significantly from that to non-degradable materials and depends strongly on the type of material. It has already been shown that CD68^+^ and CD3^+^ cells migrated into the wall of TPU grafts after one week of implantation, whereas only CD20^+^ cells were seen in ePTFE prostheses [119]. Interestingly, it was shown that polycarbonate urethane (PCU) promoted significantly less macrophage infiltration than ePTFE at 12 months of implantation [43]. TPU/TPUU implantation did not lead to any significant inflammatory reaction in short- and long-term implantation compared to autologous controls [40].

The upregulated pro-inflammatory mediators were shown to be mainly involved in inducing immune cell migration and infiltration, as well as T cell recruitment [51,64]. Moreover, in anastomotic regions of TPU/TPUU and ePTFE grafts, the regulation of the inflammatory response was mediated by the expression of anti-inflammatory proteins. Increased expression of cytokines associated with myelin/nerve fiber formation, particularly in synthetic graft samples was observed. These findings are of particular interest since it was shown before that vascular graft implantation leads to peripheral nerve injury. Nerve regeneration around the vascular implant seemed to improve graft patency due to the support of homeostasis, hemodynamic stabilization and matrix regeneration [71]. It was further observed that TPU/TPUU and ePTFE anastomoses expressed high levels of pro-angiogenic cytokines. As angiogenesis is strongly connected with inflammation, the increased expression of EG-VEGF and IGFBP-2 in the synthetic conduits was not surprising. The expression of angiogenic cytokines remained low in autologous graft anastomoses.

Based on the results in the anastomotic regions, we assumed that the PVATs of one-week autologous, TPU/TPUU, and ePTFE implants might differ in their protein and gene expression. Evaluation of graft healing showed similar gene expression profiles in the PVATs of TPU/TPUU and autologous grafts in long-term implantations [40], which was confirmed in the early healing phase. Immunostaining of isolated PVAT cells showed that PVAT from the mid-graft region of ePTFE implants expressed significantly fewer anti-inflammatory proteins like adiponectin and CD163. Furthermore, higher expression of CCR7 was observed in ePTFE PVAT. These results suggested more PVAT inflammation in ePTFE implants. It has previously been shown that the PVAT of the rat abdominal aorta contains a significant amount of pro-inflammatory cells [120], which might have been attracted more by ePTFE implants than TPU/TPUU and autologous grafts in the current study. Interestingly, ePTFE PVATs contained fewer CD105^+^ cells than TPU/TPUU and autologous PVATs. Anderson et al. (2013) showed that murine CD105^–^ mesenchymal stem cells were susceptible to adipogenic and osteogenic differentiation and suppressed the proliferation of CD4^+^ T cells [121]. CD105 is also an important marker for the proliferation, migration and differentiation of vascular endothelial cells [122,123]. The low expression of CD105 could therefore have two consequences: either a suppression of the adaptive immune response and/or limited formation of blood capillaries in the PVAT of ePTFE grafts. CD73, an anti-inflammatory mesenchymal stem cell marker, was less expressed in ePTFE compared to TPU/TPUU. Since CD73-expressing mesenchymal stem cells were essential for repair and regeneration after myocardial infarction [124] and inhibited pro-inflammatory macrophage activity [125], they may also play an important role in the healing of SDVGs. Gu et al. (2019) performed single-cell RNA sequencing and indeed showed that stem cells from PVAT contributed to vascular remodeling [126]. These cells also expressed high levels of S100A4, a protein normally expressed on fibroblasts and downregulated in the current study in PVATs of TPU/TPUU.

To evaluate the expression of inflammation and remodeling markers in PVATs, a panel of 37 genes was performed by RT-qPCR. Interestingly, many genes encoding proteins upregulated in the anastomotic regions did not show different expression patterns in PVATs from different implants. One exception was CCL2, which showed both higher protein expression levels in the anastomoses and higher gene expression in PVATs from ePTFE implants. Other genes that differed significantly in expression between graft materials were all associated with inflammation. Increased expression of CD68 in ePTFE PVATs indicated a greater presence of macrophages. Podoplanin showed increased expression in TPU/TPUU and ePTFE PVATs compared to autologous controls. Podoplanin has been shown to be upregulated during inflammation by a variety of cell types including macrophages, lymphatic endothelial cells, fibroblasts, T cells, and epithelial cells [127]. IL-1α showed a substantial increase in PVATs of ePTFE-SDVGs. Since the majority of upregulated cytokines in the anastomotic regions of TPU/TPUU and ePTFE are described as proteins regulated by IL-1, it is evident that IL-1 is a key cytokine involved in the inflammatory response to vascular graft implantation. Dinarello (2017) described the IL-1 family as factors that primarily mediate the innate immune response but also have multiple functions in adaptive inflammation [128]. In particular, IL-1α is highly expressed upon tissue injury and mediates immune cell recruitment [129]. Since PVATs from autologous and TPU/TPUU implants showed significantly increased expression of anti-inflammatory MSC markers compared to ePTFE, it is possible that MSCs reduced the IL-1α gene expression in these conditions, which was not the case with ePTFE grafts. CD206 has been described as an anti-inflammatory macrophage marker of adipose tissue [130,131]. Similar to the anastomotic regions, the expression of anti-inflammatory genes could indicate a regulation of the strong inflammation around ePTFE implants.

The current study has shown that the early healing response to different vascular graft materials varies significantly depending on the material used. It has been demonstrated that ePTFE elicits a significantly stronger pro-inflammatory immune response than autologous and biodegradable TPU/TPUU grafts. Previous studies have shown that ePTFE exhibits continuous expression of pro-inflammatory cytokines over prolonged implantation periods [42,43], which can lead to chronic inflammation and wounding. We were able to show that this persistent inflammation occurs as early as 24 h after implantation. Long-term graft patency and prevention of chronic inflammation require an early phenotype switch from pro-inflammatory (M1) to anti-inflammatory (M2) macrophages in the early phase of implantation [132,133]. In addition, a suitable pore size and porosity to allow transmural migration of endothelial cells and vascularization of the conduit are important to prevent thrombosis [[134], [135], [136]]. Negatively charged, hemolytic or immunogenic surfaces can lead to thrombogenic protein adsorption during implantation [137]. Therefore, adequate protein adsorption is another key factor for graft survival, as it supports the endothelialization [138].

To date, there is no synthetic SDVG that fulfills all these requirements. New fabrication technologies such as 3D bioprinting [139,140], as well as immunomodulation and biofunctionalization techniques [[141], [142], [143]] can bring us closer to an ideal, patent SDVG for clinical application.

## 5 Limitations

The current study showed the early healing phase of different vascular graft materials. A major limitation is the realization of the *in vivo* experiments in a rat model since inflammation and the activation of different immunological pathways might have not reflected the situation in human patients [144]. Nevertheless, the present study adds a substantial knowledge to the understanding of the early healing phase of different SDVG materials.

Considering the delay in the inflammatory response to TPU/TPUU grafts, a longer time point, for example two weeks, could have been chosen to investigate the resolution of inflammation in these grafts in more detail.

Furthermore, disease models should be used to evaluate the healing process of vascular grafts in preclinical studies, since clinical application usually involves multimorbid patients. Ardestani et al. (2020) described a new apolipoprotein E-knockout rat model that develops signs of atherosclerosis, as evidenced by increased plasma cholesterol, low-density lipoprotein and triglyceride levels when fed a Western diet. These rats also showed calcification and an accumulation of macrophages and foam cells in the aortic arch [145]. Another option would be to use diabetic BB Wistar rats [146,147], since the metabolic and cardiovascular systems interact closely to maintain homeostasis [148].

## 6 Conclusion

In conclusion, the data of the current study suggest a prolonged initial inflammatory response and suppression of the activation of immune cells of the adaptive immune system in synthetic SDVGs. Surprisingly, the inflammatory response was delayed in TPU/TPUU by several days. The immune response to autologous grafts remained significantly low between one and seven days of implantation. However, both TPU/TPUU and ePTFE showed increased cytokine expression in the anastomotic regions compared to autologous implants, suggesting a higher inflammatory response and remodeling at the site of tissue injury. Significant inflammation was observed in the PVAT of ePTFE grafts, which could potentially lead to thrombosis, atherosclerosis and intimal hyperplasia in long-term implantation. Therefore, new innovative materials and therapeutic strategies are crucial to address the inadequate early healing response that currently limits graft performance. A particular focus should be placed on understanding the role of PVAT in vascular graft healing.

## CRediT authorship contribution statement

**Sabrina Rohringer:** Writing – original draft, Methodology, Investigation, Formal analysis, Data curation. **Sophie Johanna Specht:** Writing – review & editing, Investigation. **Anna-Maria Schmitt:** Writing – review & editing, Investigation. **Selin Topcu:** Writing – review & editing, Investigation. **Karl Heinrich Schneider:** Writing – review & editing, Resources, Investigation. **Marjan Enayati:** Writing – review & editing. **Christian Grasl:** Writing – review & editing, Methodology, Investigation. **Katharina Ehrmann:** Writing – review & editing, Resources. **Stefan Baudis:** Writing – review & editing, Resources. **Herbert Kiss:** Writing – review & editing, Resources. **Heinrich Schima:** Writing – review & editing, Resources, Data curation. **Bruno Karl Podesser:** Writing – review & editing, Resources. **Helga Bergmeister:** Writing – original draft, Validation, Supervision, Methodology, Investigation, Funding acquisition, Conceptualization.

## Data statement

The research data is stored in a Mendeley data repository and is accessible via following link: https://data.mendeley.com/preview/8s8kn4c9j5?a=cc5a1d8e-8a50-465a-8837-e60a4e67ba1d.

## Declaration of generative AI and AI-assisted technologies in the writing process

During the preparation of this work the authors used www.deepl.com in order to improve language and readability. After using this tool, the authors reviewed and edited the content as needed and take full responsibility for the content of the publication.

## Funding sources

The study was partially funded by the Ludwig Boltzmann Institute for Cardiovascular Research. This study is supported by the HORIZON-EIC-2022-PATHFINDERCHALLENGES-01 project Blood2Power (grant agreement number 101115525) funded by the European Union.

## Declaration of competing interest

The authors declare that they have no known competing financial interests or personal relationships that could have appeared to influence the work reported in this paper.

## Data Availability

Data will be made available on request.
