# Supplementary material for: Autologous and synthetic vascular grafts trigger different host responses in the anastomotic regions and in the perivascular adipose tissue during the early healing phase
Source: Mater Today Bio. 2025 Jul 15;33:102089. doi: 10.1016/j.mtbio.2025.102089 (PMC12284494; doi:10.1016/j.mtbio.2025.102089)
Supplement: Multimedia component 1 [file mmc1.docx]

**Supplementary information**

**Autologous and synthetic vascular grafts trigger different host responses in the anastomotic regions and in the perivascular adipose tissue during the early healing phase**

Sabrina Rohringer^1,2,3^, Sophie Johanna Specht^1,2,3^, Anna-Maria Schmitt^1,2,3^, Selin Topcu^1,2,3^, Karl Heinrich Schneider^1,2,3^, Marjan Enayati^1,2,3^, Christian Grasl^2,4^, Katharina Ehrmann^3,5^, Stefan Baudis^3,5^, Herbert Kiss^6^, Heinrich Schima^2,4^, Bruno Karl Podesser^1,2,3^, Helga Bergmeister^1,2,3^


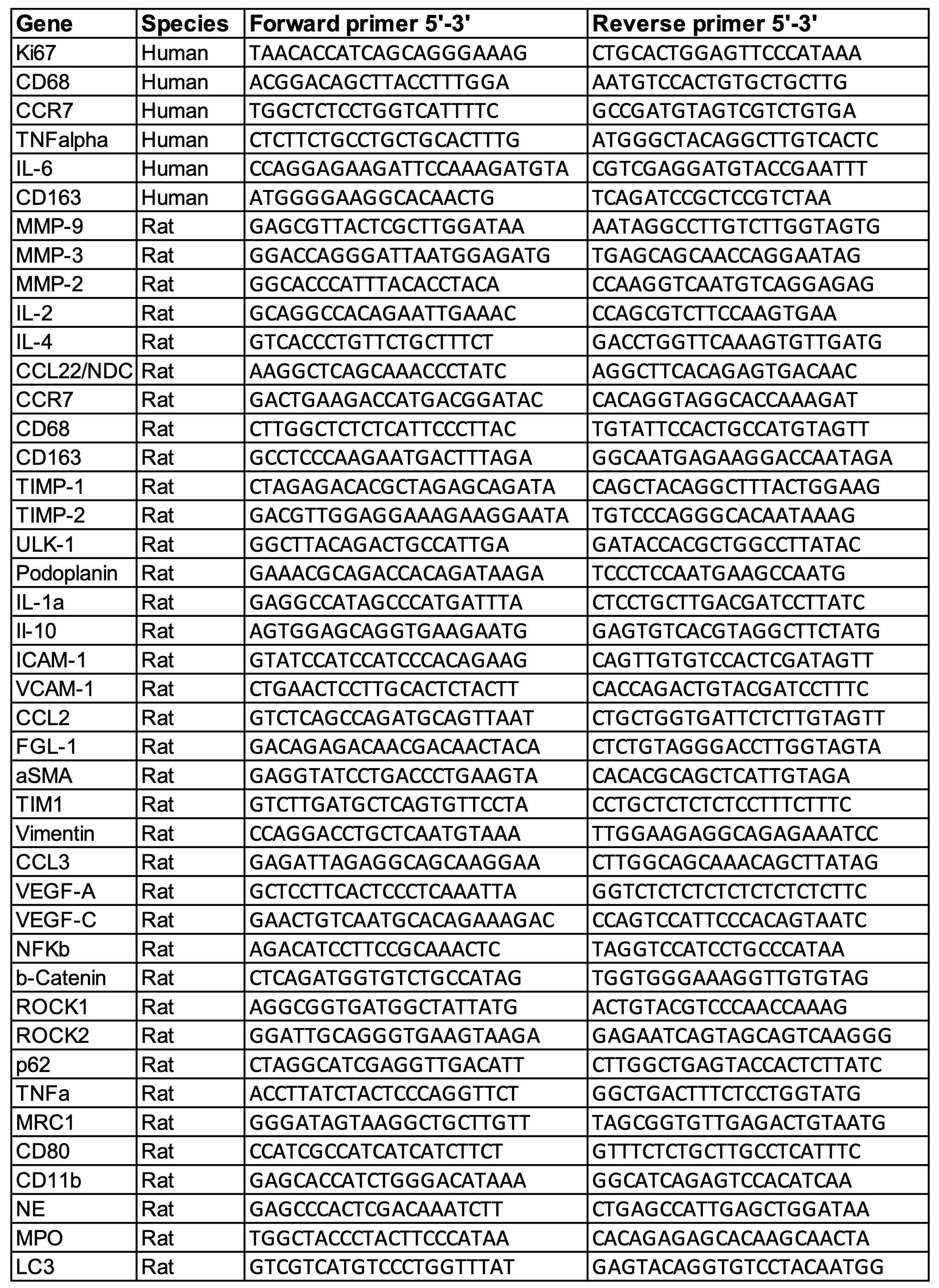


Supplementary Figure S1: Primer list for RT-qPCR


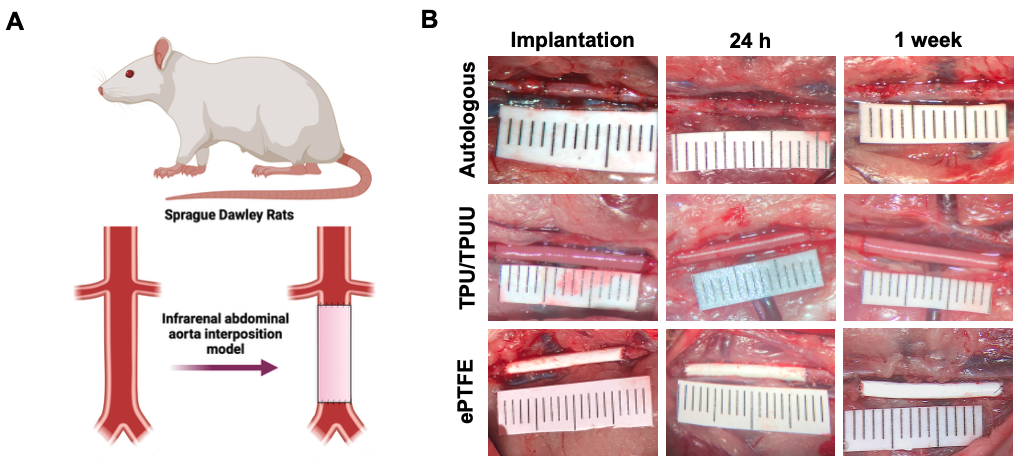


Supplementary Figure S2: In vivo implantation. Autologous, TPU/TPUU and ePTFE SDVGs were implanted into the infrarenal aorta of male Sprague Dawley rats (A). Grafts were retrieved after 24 h and one week. None of the grafts showed severe inflammation on the macroscopic level (B).


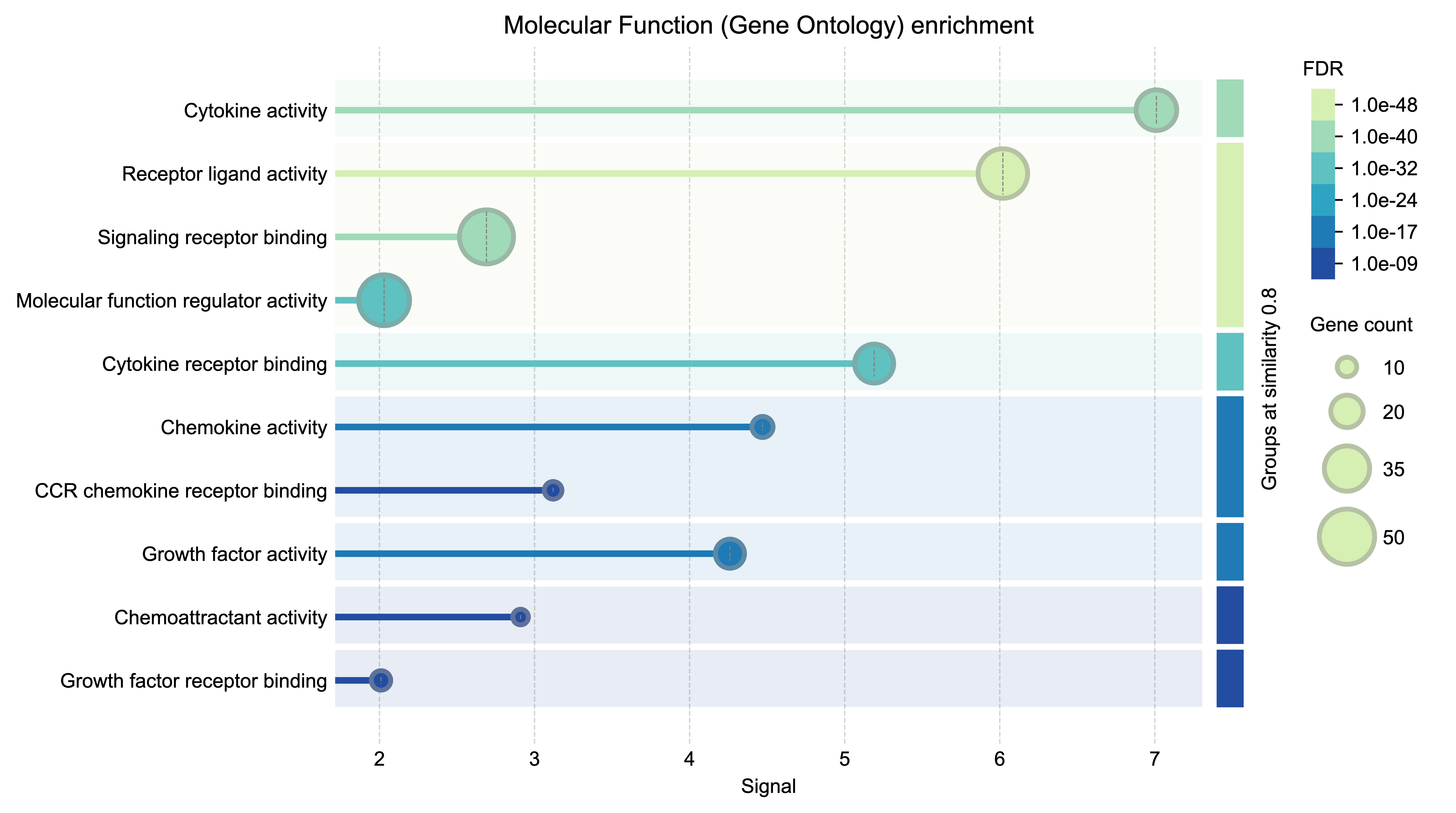


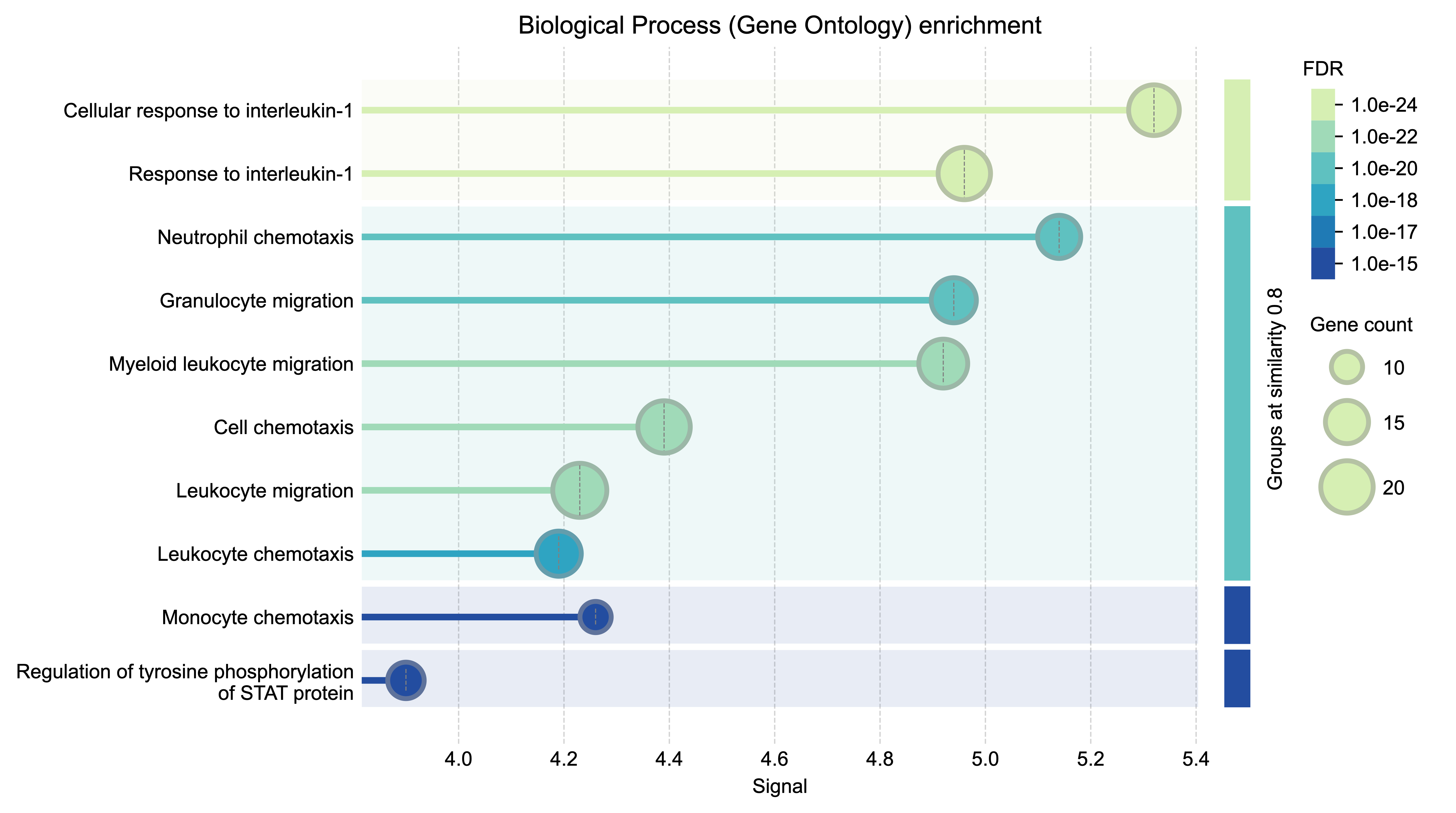


Supplementary Figure S3: Cytokine arrays of anastomotic regions. Clustering of the analyzed cytokines revealed that the majority of cytokines is involved in regulating of molecular function and cytokine activity, receptor ligand bindingand interleukin-1 pathways.
